# Can Churches Play a Role in Combating the HIV/AIDS Epidemic? A Study of the Attitudes of Christian Religious Leaders in Madagascar

**DOI:** 10.1371/journal.pone.0097131

**Published:** 2014-05-13

**Authors:** Jerry S. Rakotoniana, Jean de Dieu M. Rakotomanga, Hubert Barennes

**Affiliations:** 1 Institut Francophone pour la Médecine Tropicale, Vientiane, Vientiane, Lao PDR; 2 Institut National de Santé Publique et Communautaire (INSPC) Befelatanana, Antananarivo, Madagascar; 3 Agence Nationale de Recherche sur le VIH et les Hepatites, Phnom Penh, Cambodia; 4 INSERM, ISPED, Centre INSERM U897-Epidemiologie-Biostatistique, Bordeaux, France; 5 Epidemiology Unit, Pasteur Institute, Phnom Penh, Cambodia; Massachusetts General Hospital, United States of America

## Abstract

**Introduction:**

Churches occupy an important social and cultural position in Madagascar. The sexual transmission of HIV raises controversies about the role that Churches can play in preventing HIV/AIDS. This cross-sectional survey investigated recommendations by religious leaders for condom use and other preventive strategies in the context of international guidelines.

**Methods:**

A questionnaire was self-administered to a random sample of religious leaders. The questions related to preventive methods against HIV/AIDS such as: condom use, marital fidelity, sexual abstinence before marriage, and HIV-testing. Associations with recommendations for condom use were evaluated using univariate and multivariate logistic regression analyses.

**Results:**

Of 231 religious leaders, 215 (93.1%) were willing to share their knowledge of HIV/AIDS with their congregations. The majority received their information from the media (N = 136, 58.9%), a minority from their church (N = 9, 3.9%), and 38 (16.4%) had received prior training on HIV. Nearly all (N = 212, 91.8%) knew that HIV could be sexually transmitted though only a few (N = 39, 16.9%) were aware of mother-to-child transmission or unsafe injections (N = 56, 24.2%). A total of 91 (39.4%) were willing to, or had recommended (N = 64, 27.7%), condom use, while 50 (21.6%) had undergone HIV testing. Only nine (3.9%) had ever cared for a person living with HIV/AIDS (PLHIV). Multivariable logistic regression shows that condom use recommendations by religious leaders were negatively associated with tertiary level education (OR: 0.3, 95% CI 0.1–0.7), and positively associated with knowing a person at risk (OR: 16.2, 95% CI 3.2–80.2), knowing of an ART center (OR: 2.6, 95% CI 1.4–4.8), and receiving information about HIV at school (OR: 2.6, 95% CI 1.2–5.6).

**Conclusions:**

Malagasy church leaders could potentially become key players in HIV/AIDS prevention if they improved their knowledge of the illness, their commitment to international recommendations, and extended their interaction with people most at risk.

## Introduction

Worldwide, various prevention and treatment strategies have been effective in reducing the extent of the HIV epidemic. The focus has been on high risk groups and has included prevention programs such as: behavior change, condom use promotion, HIV testing, safe blood supply, harm-reduction efforts for intravenous drug users, and male circumcision.

Religious leaders could potentially make a considerable contribution to HIV/AIDS prevention. Due to their status in society they have been progressively included as essential contributors in the fight against HIV [1], [2]. The early involvement of religious leaders in combating AIDS in Senegal and Uganda has been described as a particularly important, and a replicable factor in HIV/AIDS reduction elsewhere [3], [4].

In the context of the HIV epidemic, groups of Christian and Islamic faith healers (different from traditional healers because of their reliance on religion) emerged in sub-Saharan countries [5]. Since they had the potential for benefiting or exacerbating efforts to fight the HIV/AIDS epidemic, such faith healers were suggested as constituting a third therapeutic system that could coexist with well-documented biomedical and traditional healers. The response of religious leaders to HIV/AIDS has been mixed in developed countries [6].

Churches have always played a dominant role in the social life, welfare, education and politics of Madagascar. Some issues, particularly those related to sex and condom use, the lynchpin of international HIV prevention strategies, may conflict with church doctrines [7]. Madagascar churches elaborated their own action plans in response to the Madagascar Action Plan 2007–2012 to combat HIV/AIDS [8]. The plan required proper communication with congregations to bring about behavior change and social transformation. Active involvement of community leaders and their organizations was sought. While the importance of involving religious leaders in HIV/AIDS prevention has been established in other countries, estimating their real contribution in Madagascar remains speculative.

Amongst the Malagasy, the dominant ethnic group in Madagascar, the low use of condoms remains a major concern. The use of modern contraceptives by women is infrequent, abortions are frequent, and condoms are often limited to preventing pregnancy during fertile days [9], [10]. A survey conducted at social venues of 2,982 individuals showed that only 28% of men and 41% of women reported using condoms during their last sexual activity with a new partner [11]. Condom use is even lower among youth with less than 15% having used one during their last intercourse with a regular partner [12]. Among students, despite the increasing number of those known to be HIV-infected, only 5.7% reported consistent condom use [13].

The present study aims to evaluate the tendency of church leaders towards recommending condom use for preventing the sexual transmission of HIV in the context of the national HIV plan, the ABC strategy (abstain, be faithful and use condoms) [8], and international recommendations [14].

## Materials and Methods

### Ethics

The Francophone Institut of Tropical Diseases and the Malagasy Ethical Committee gave approval to conduct the survey. Informed oral consent was obtained from the participants.

### Study Context

In Madagascar 68% of the population (20 million inhabitants: 45% under 15 years) lives below the poverty line of USD 1.25 per day [14]. Annual per capita income was USD 980 in 2011. The literacy rate for men and women is 68% and 66%, respectively. Since the first case was reported in 1987, Madagascar has been relatively protected from the HIV epidemic as prevalence among adults aged 15 to 49 is estimated at 0.30% [0.20%–0.40%] [16], [15]. Nevertheless, the high rate of sexually transmitted infections (STI) in the at-risk and rural populations could fuel an HIV/AIDS epidemic [16]–[20]. In addition, risky sexual behaviors such having multiple partners and paid sex are common, and the rate of condom use is low [9], [21], [24]. Other risk factors include poverty, limited access to health and social services, and limited knowledge and understanding of HIV/AIDS [22], [23].

Christians make up almost 50% of the population, followed by animists (43%) and Muslims (7%). There are 120 religious entities registered with the Ministry of the Territory. The Malagasy Council of Christian Churches (FFKM), founded in 1980, is the umbrella organization for the country’s four principal Christian denominations: Roman Catholics (4 million members), Reformed Protestant Church of Jesus Christ in Madagascar (FJKM 2 million members), Malagasy Lutheran (FLM) (3 million members), and the Anglicans (30,000 members). The oldest of the Lutheran congregations was founded in the early 19^th^ century. Its success is due, in part, to an indigenous revivalist movement, known as Fifohazana, that has worked through its church since the early 20th century. The FFKM is a traditional leader in education and a key player in a broad range of social welfare activities including hospitals, dispensaries and community health projects (with special initiatives that cover child survival), family planning and HIV/AIDS prevention. Additionally, the FFKM has served as a mediator, bringing together antagonistic political factions.

### Study Design and Procedures

This cross-sectional survey was conducted from February to June 2009 in Antsirabe, the second largest city in Madagascar, with a population of 250,000. The city has 32 religious denominations, 126 churches, 6 clinics and hospitals, and 22 primary and 24 secondary schools. Using a random numbers table, two churches were selected from each denomination with more than 20 churches, and one church from those with less than 20.

#### Inclusion criteria

**Figure 1:**
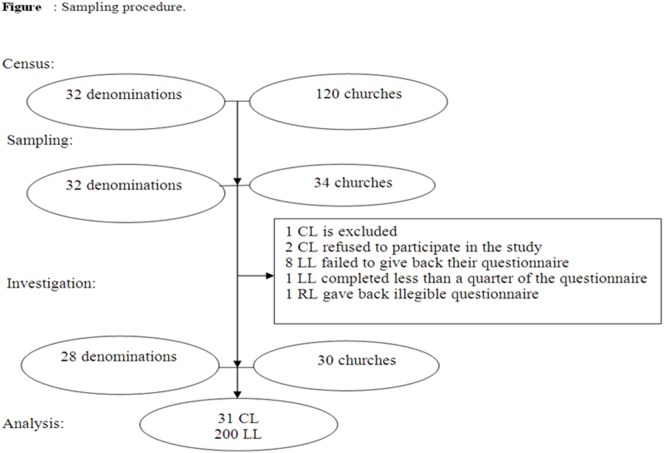
Flow chart of the survey of religious leaders in Madagascar.

After informed consent, church leaders (CL) and lay leaders (LL) who had been working in the church prior to 2009 were included, if they were able to dedicate one hour to the survey (Figure 1).

#### Survey questionnaire and procedure

The leaders of selected churches were gathered in a room allocated for the survey. The objectives of the survey were explained to them and questions, if any, were answered. A questionnaire was self-administered. It was adapted from another unpublished survey carried out amongst Malagasy youth by FFKM in 2004. It consisted of 56 questions divided into four sections: (1) a socio-demographic section, (2) a section dealing with knowledge of HIV and its prevention (abstaining from sexual intercourse before marriage (ASIBM), marital fidelity (MF), use of condoms, management of STI-AIDS, (3) a section on interviewee willingness to share knowledge of HIV/AIDS, recommending condom use and other prevention means, willingness to be tested for HIV and to engage with People Living with HIV/AIDS (PLHIV), and finally (4) a section on practices. This last section explored religious leaders’ prior experience of counseling people on HIV prevention and testing, and their previous contact with PLHIV.

The willingness to recommend condom use was used as a primary outcome. Practices related to recommendation of condom use, and other preventive strategies were used as secondary outcomes.

#### Sample size

Using Stata Version 8 (Stata Cooperation, College Station, TX), a required sample size of 197 people was established, based on an estimate of 50% rate of condom use recommendations by LL, with 12% precision, alpha = 0.05, power 90% and 10% anticipation for drop-outs or refusals. The final sample size was rounded off to 200 people, allowing for an estimate of condom use recommendations ranging from 42% to 57%. We enrolled one church leader for each selected Church.

### Data Management and Analyses

Data were entered in Epidata freeware (www.epidata.dk, Odense, Denmark) and cross-checked against original data sheets. Analyses were carried out with Stata software. Chi^2^ or Fisher’s exact test were used to assess associations between categorical variables as appropriate, and Student’s t-test for two normally distributed continuous variables. P≤0.05 was considered significant. Associations with the willingness to recommend condom use by religious leaders were initially measured using univariate analysis for different variables (age, sex, years in the position, education, specific training on HIV, information source, knowledge on HIV and STI transmission and prevention, previous contact with PLHIV, Church denomination). Multivariate analyses was then conducted introducing into the model the variables significantly associated with condom recommendations with *p*-values <0.2. We then followed a back-step selection procedure using odd ratios to leave only those with a *p*-value <0.05 in the final model.

## Results

### Socio-demographic Characteristics

A total of 231 people (31 CL and 200 LL) were enrolled in the survey (Figure 1).

Religious leaders represented 28 different Christian denominations (Table 1) (details can be found in Table S1). Six denominations were represented by more than 10 people (Malagasy Lutheran Church: 21, Catholics: 20, Savior Jesus: 15, Seventh-Day Adventist Church: 12, Holy Spirit Revival Church of Madagascar: 12, Rhema: 11). Two denominations had a condom recommendation rate over 50% (Catholics: 55%, Revival Church: 100%). The average rate for recommending condom use did not differ between Catholics and other religious groups (11/21, 52.4% vs. 80/210, 38.1%, p = 0.20, respectively). The social characteristics of religious leaders are shown in Table 2 and 3. CL and LL were similar in age, education and average seniority in the church but there were more women LL than CL, (N = 85, 42.5% vs. n = 3, 9.7%, p<0.000, respectively). About one-quarter of religious leaders led youth groups in the church, and/or were teachers and 38 participants (16.4%) had received prior training on HIV.

**Table 1:** List of Christian denominations represented by over 5 religious leaders.

Denominations*	Leaders included	Will recommend condom
	N = 231	N = 91 (%)**
Malagasy Lutherian Church	21	8 (38.1)
Roman Catholic Church	21	11 (52.4)
Rhema	15	3 (20.0)
Savior Jesus	15	3 (20.0)
Seventh-Day Adventist Church	12	4 (33.3)
Holy Spirit Revival Church of Madagascar	12	12 (100.0)
Church of Jehovah, the Setter of Sabbath	10	3 (30.0)
New Protestant Church of Madagascar	10	3 (33.3)
Jesus Christ Church of Madagascar	9	4 (44.4)
Sons of Reconciliation	9	1 (11.1)
Anglican Church	8	5 (62.5)
Church of The Revelations	8	3 (37.5)
God’s Assembly	7	5 (71.4)
Grace Church	7	1 (14.3)
Malagasy Church of Reed and Bricks	6	1 (16.7)
Native Evangelic Mission of Madagascar	6	5 (83.3)
Biblical Baptist Church	6	1 (16.7)
Jehovah’s Witnesses	6	2 (33.3)
Yawoshua New Convenant	6	1 (20.0)
The church of JC of latter-day Saints	6	1 (16.7)
Less than 5 leaders enrolled***	31	14 (45.1)

*4 did not answer the question about denomination,

**16 did not answer this question.

***New Life Baptist Church (N = 5, 4: 80%), Evangelical Church (N = 5, 2∶40%), Revival Church of Jesus’ Disciples (N = 5, 1∶20%), Denomination unknown (N = 4, 3∶75%), Jesus Winner and Union of the Pentecostals and Jesus’ Wife Community (N = 3, 1∶33.3%), Free my People (N = 2, 1∶50%), Baptist Church of Mamorivokatra (N = 1, 0∶0%).

**Table 2:** Socio-demographic characteristics of religious leaders in Madagascar.

Study population characteristics	Total
	n = 231 (%)
**Socio-demographics**	
Age (years) Median (IQR)	42.8 (34.5–51.8)
Male gender	143 (61.9)
Married	200 (86.6)
Non-Catholic	210 (90.1)
Member of Malagasy Council of Churches*	60 (26.0)
**Position in church**	
Religious leader	31 (13.4)
Lay leader	200 (86.6)
Over 5 years in the church	135 (58.4)
**Main occupation**	
Low skills**	51 (22.1)
Civil servant/entrepreneur	46 (19.9)
Shopkeeper	42 (18.2)
Unemployed	41 (17.8)
Priest	31 (13.4)
**Education** ***	n = 216
Primary	12 (5.5)
Secondary	124 (57.4)
Tertiary (university)	80 (37.0)
**Trained in HIV**	38 (16.5)

Numbers and (percentages). Mean and [95% confidence interval].

*FFKM is the Malagasy Council of Christian Churches.

**Low skills: Farmers, manual laborers, Unemployed includes no official position, retired people, housewives.

***15 missing responses.

**Table 3:** Religious leaders’ knowledge, attitude and practice towards different condom use recommendations and other strategies of campaign against HIV-AIDS.

	Condom use recommendation		Univariate analysis			Multivariate comparison		
	Willing to	Not willing to	Crude OR	95%CI	p	Adj. OR	95%CI	p
	N = 91 (%)	N = 140 (%)						
**Socio-demographics**								
Age >40	58 (63.7)	86 (61.4)	1.1	0.6–1.9	0.7	NI		
Males	57 (62.6)	86 (61.4)	1.0	0.5–1.8	0.8	NI		
Married	79 (86.8)	121 (86.4)	1.0	0.4–2.7	0.9	NI		
Member of FFKM	27 (29.7)	33 (23.6)	0.7	0.3–1.3	0.3	NI		
Catholic*	11 (12.1)	10 (17.1)	1.7	0.6–4.9	0.2	NS		
**Position in church**								
Lay leader	73 (80.2)	127 (90.7)	1 (Ref.)					
Church leader*	18 (19.8)	13 (9.3)	0.4	0.1–0.9	0.02	NS		
Worked <5 years	29 (31.8)	88 (62.9)	1 (Ref.)					
>5 years*	62 (68.1)	52 (37.1)	3.6	1.9–6.5	<0.001	NS		
**Education** * **^,^** µ								
Secondary	51 (56.1)	73 (52.1)	1 (Ref.)			NS		
Primary	9 (9.9)	3 (2.1)	0.2	0.0–1.0	0.02	NS		
Tertiary (university)	28 (30.8)	52 (37.1)	1.2	0.6–2.4	0.3	0.3	0.1–0.7	0.01
**Sources of information** µµ								
Media*	48 (52.8)	88 (62.9)	1 (Ref.)					
Awareness campaign	20 (22.0)	24 (17.1)	0.6	0.3–1.3	0.2	NS		
School*	23 (25.3)	21 (15.0)	0.4	0.2–1.0	0.04	2.6	1.2–5.6	0.02
Training	20 (22.0)	18 (12.9)	0.4	0.2–1.0	0.06	NS		
HIV Health Center	12 (13.2)	10 (7.1)	0.4	0.1–1.2	0.08	NI		
Church	5 (5.5)	4 (2.9)	0.4	0.0–2.1	0.2	NI		
Others	5 (5.5)	6 (4.3)	0.6	0.1–2.8	0.4	NI		
Word of mouth	4 (4.4)	4 (2.9)	0.4	0.0–2.1	0.2	NI		
**Knowledge of HIV preventive strategies**								
Blood transfusion test*	83 (91.2)	112 (80.0)	1.6	0.8–2.9	0.09	NS		
HIV testing and counseling*	65 (71.4)	85 (60.7)	1.3	0.7–2.3	0.3	NS		
Marital fidelity	61 (67.0)	85 (60.7)	1.6	0.8–2.9	0.09	NS		
Condoms	50 (55.0)	43 (30.7)	2.7	1.5–4.9	<0.001	NI		
ASIBM*	29 (31.9)	27 (19.3)	1.9	1.0–3.7	0.02	N/A		
Safe management of SI	23 (25.3)	33 (23.6)	1.0	0.5–2.1	0.7	NI		
PMTCT*	25 (27.5)	14 (10.0)	3.4	1.5–7.5	<0.001	NS		
**Knowledge about HIV patients and treatment centers**								
Does not know any PLHIV	82 (90.1)	133 (95.0)	1 (Ref.)					
Knows PLHIV*	8 (8.8)	7 (5.0)	0.5	0.1–1.7	0.2	16.2	3.2–80.2	0.001
Knows a HIV testing center*	69 (75.8)	86 (61.4)	0.7	0.4–1.1	0.2	NS		
Knows one ART center*	63 (69.2)	72 (51.4)	0.7	0.4–1.1	0.1	2.6	1.4–4.8	0.004
**Attitudes towards patients and HIV preventive strategies**								
Willing to help PLHIV	90 (98.9)	133 (95.0)	4.7	0.5–215	0.1	NS		
- to share knowledge on HIV	84 (92.3)	131 (93.6)	0.8	0.2–2.7	0.7	NI		
Willing to advise ASIBM	91 (100.0)	129 (92.1)	N/A			NI		
- at church	78 (85.7)	106 (75.7)	1.9	0.9–4.2	0.06	NS		
Willing to recommend MF	90 (98.9)	137(97.9)	1.9	0.1–100	0.5	NI		
- at church	6 (6.6)	10 (7.1)	0.9	0.2–2.9	0.8	NI		
**Practices**								
Already had an HIV test	25 (27.5)	25 (17.9)	3.0	1.2–7.1	0.005	NS		
Already recommended condoms	48 (52.8)	16 (11.4)	8.6	4.2–17–9	<0.001	NA		

Numbers and (percentages). Mean and [95% confidence interval].

CI: Confidence interval. OR: Odds Ratio. Ref.: reference. Adj.: adjusted. NI: non-included in multivariate analysis. NS: non-significant. N/A: Non-applicable. FFKM: Malagasy Council of Christian Churches. ART: antiretroviral treatment. PMTCT: prevention of mother to child transmission. PLHIV People living with HIV/AIDS. ASIBM: sexual abstinence before marriage. MF: marital fidelity. SI: sharp instruments.

*Variables included in the multivariate analyses. ORs are shown for variables forced into the model,

µ Of 88 and 128 participants, respectively.

µµ Multiple answers possible.

### Knowledge

The majority of religious leaders (N = 213, 92.2%) considered HIV a lethal and incurable disease. Religious leaders got their information primarily from the media (N = 136, 58.9%), awareness campaigns or at school (N = 44, 19.1%), and only nine (3.9%) from their church. A total of 38 (16.4%) had received prior training on HIV (Table 3). Of 231, 212 (91.8%) knew that AIDS was an illness transmitted by sexual intercourse, 195 (84.4%) by unsafe blood transfusions or unsafe injections, but only 39 (16.9%) were aware of mother-to-child transmissions. Overall CL had much better knowledge of preventive measures than LL regarding recommendations for condom use (N = 22, 71.0% versus N = 70, 35.0%; p = 0.001), marital fidelity (N = 26, 83.9% versus N = 113, 56.5%, p = 0.004), and ASIBM (14, 45.2% versus 42, 21.0%, p = 0.003). Fifteen leaders (6.5%) knew a PLHIV while 155 (67.1%) could indicate a HIV testing center and 135 (58.4%) an ART center.

### Attitudes and Practices

A total of 215 leaders (93.1%) were willing to share their knowledge of HIV-AIDS and 91 (39.4%) to recommend condoms for prevention of HIV. Of 220 who answered the question, 83 (37.7%) would impose conditions on talking about condoms. The main conditions were that information should be associated with ASIBM and MF (N = 22, 26.5%), restricted to people most at risk (N = 29, 34.9%) or married (N = 14, 16.8%), or restricted to contraception (N = 6, 7.2%). Only 16 leaders (6.9%) would allow somebody to conduct a session on condoms in church, and only two (0.8%) would do it personally. CL showed a higher willingness to recommend condoms than LL (N = 18, 58.1% versus N = 73, 36.5%, p = 0.001, respectively). The willingness to/not to recommend condom use did not differ among religious leaders according to previous HIV training (N = 20, 22% vs. 18, 12.1%, p = 0.06). Reasons for the reluctance to educate on condoms in the church were that officially recommending their use could be considered as encouraging sexual permissiveness (N = 67, 29.0%), or that condom use was ineffective 38 (16.5%).

A total of 223 (96.5%) were willing to help PLHIV. The main motives for helping were compassion (N = 106, 45.9%), moral duty (N = 64, 27.7%) and to decrease HIV transmission (N = 60, 26.0%). However, only 15 (6.5%) had had contact with PLHIV. Lay leaders had more frequent contact with PLHIV than church leaders (N = 8, 25.8% vs. n = 7, 3.8% p = 0.04) and advised more frequently people at risk to use condoms than CL (N = 28, 71.8% vs. N = 11, 28.2%, p = 0.04).

Less than a quarter of participants (N = 50, 21.7%) had personally undergone an HIV test. The primary reasons given for HIV testing was to “set an example to their community (64.0%)” rather than prevention (13.0%) or for checking their serological status (26.0%).

The practice of condom recommendation (N = 64, 27.7%) was less frequent than the willingness to recommend (N = 91, 39.4%, p = 0.01) and 16 participants (6.9%) did not answer this question. The main reasons for recommending condoms in the past were: recommendation to relatives (N = 47, 20.3%), recommendation as contraceptive (N = 31, 13.4%), HIV prevention (N = 24, 10.3%), or for people at risk (N = 15, 6.4%). In the group not willing to recommend condom use, a few participants (N = 16, 11.4%) stated that they had already recommended it.

The multivariate regression analysis shows that recommendations for condom use were independently associated with four factors. It was negatively associated with a tertiary education (OR: 0.3, 95% CI 0.1–0.7), and positively associated with knowing a person at risk (OR: 16.2, 95% CI 3.2–80.2), knowing of an ART center (OR: 2.6, 95% CI 1.4–4.8), and receiving information on HIV from school (OR: 2.6, 95% CI 1.2–5.6) (Table 3).

## Discussion

This survey highlights the opinions of Christian religious leaders in the context of the low prevalence of HIV but high prevalence of STI in Madagascar. Surveys studying the attitude of religious leaders to HIV in a traditionally religious country are uncommon. The results of this survey show that most Malagasy Christian leaders are willing to play a role in informing the population about HIV/AIDS. It clearly underlines the limitations on the willingness of religious leaders to get involved. The poor commitment of religious leaders to international recommendations on condom use may question the effectiveness and impact of their collaboration in any campaign against HIV. The majority of religious leaders restricted themselves to acting mainly within doctrinal teachings and interactions between them and PLHIV were also infrequent.

The majority of religious leaders quoted media as the major source of information. This may have been because the media had exposed a number of blood transfusion scandals in the 1980–1990s, thus raising awareness that HIV could be passed on through blood transfusions [25]. Religious leaders were able to distinguish between media and awareness campaigns. The awareness campaign, which was supposed to provide more robust information on the disease, proved insufficient compared to media influences. Due our study design, the role of information sources could not be investigated further. This suggests that the media would have to be used more effectively to improve the general knowledge of the population.

Few religious leaders (N = 38, 16.5%) received prior training and this was probably not sufficient to significantly impact the rate of condom recommendations. This suggests that training curricula and post training practices need to be re-evaluated in agreement with international HIV prevention programs in order to improve effectiveness.

Interestingly, condoms as a preventive tool were reported by 40.3% of the religious leaders and surpassed ASIBM (24.2%). However, the level of recommendation (39.4%) was low compared to ASIBM (95.2%) and MF (98.3%) recommendations. These results were consistent with other surveys among religious leaders in Malawi which showed that although these people may effectively promote A (abstinence) or B (be faithful), many prohibit or at least fail to endorse C (condom use) [2], [7].

Of interest for condom promotion, was the fact that some participants (16, 11.4%) of the group who declared not willing to recommend condom use reported that they had already recommended it. Amongst the Malagasy population the low use of condoms remains a major concern. Several studies support this crucial issue [9], [10], [12]. In Madagascar negative perceptions of contraceptive methods are associated with misinformation and social opposition to their use mainly from male partners [10]. The reasons for condom use are highly related to accessibility, parental support for condom use, patterns of risky sexual behavior, and the perception of condom effectiveness in family planning [12]. The reasons for non-use of condoms were steady relationships, the perception that condoms were useful only during ovulation periods, and the decrease of pleasure [21]. The low use of condoms remains a cause of concern, not only for HIV prevention but also in reducing the high prevalence of syphilis, other STI’s, and abortions [16], [17]. The involvement of religious leaders is crucial regarding these issues as they are considered the toughest impediment to condom use [12].

Another interesting finding was the fact that CL showed a higher willingness to recommend condoms than LL. However, they had less contact with people at risk than LL. A recent examination of condom use in Malawi documents a host of objections but found no evidence that religion impedes condom use [7]. Furthermore, LL whose religious leaders accepted condoms, were significantly more likely to report using them personally. Therefore, well-respected religious leaders in Madagascar could influence condom use by encouraging LL within their religious communities. HIV/AIDS campaigns managers in Madagascar could consider targeting CL to enhance the adoption of condom use recommendations.

ASIBM and marital fidelity, two church teachings, were the primary prevention measures advocated by the religious leaders, although marital fidelity was infrequently advocated in churches. The reason for not introducing this matter in the religious teaching is unclear and it was not addressed further in this study. However, persistently high levels of STI in Madagascar indicate that these current prevention strategies are inadequate. In fact, one could question the religious leaders’ influence on the general behavior of the population and more specifically whether they were able to reach the target groups for HIV/AIDS prevention. These points are questioned by various reports from Tanzania, Malawi and South Africa [1], [26]–[28]. These reports showed that risky sexual behavior was less prevalent among members of strict religious minority groups and among those reporting high levels of religious commitment overall. The literature and this study suggest that religious leaders should consider enhancing their influence with the population most at risk.

This study showed that the response of Christian religious leaders to condom use varied and was related to their position and denomination. Interestingly, a positive result from this study was that some religious leaders were willing to accept HIV testing and specific condom sessions in their churches. It showed that some religious leaders could extend their traditional role to health promotion activities as documented in other countries [2]. HIV/AIDS programs may consider involving more of these kinds of leaders in their interventions.

HIV-related stigma is an important barrier to prevention messages, but was not specifically investigated in this survey. The fact that over 95% of religious leaders were ready to help, and half of the few who knew a PLHIV had been helpful, indicated a positive commitment but gave no clue regarding stigma among religious leaders in Madagascar. In other countries, some religious leaders were reported to exacerbate the situation. For example, in Tanzania, a survey revealed strong evidence that shame-related HIV stigma was strongly associated with religious beliefs such as that illness was “a punishment from God”, or that PLHIV had not “followed the Word of God” [29].

Most religious leaders were ready to help PLHIV. This shows that involving religious leaders could provide additional benefits related to compassion. For example, in Tanzania, PLHIV reported that prayer gave them hope, and for some, that prayer supported their adherence to medication [28].

The final multivariate analysis showed that lower education was related to a higher rate of condom use recommendations, which could be questioned. This result is not consistent with the result of the Demographic and Health Survey (DHS) 2008–2009 [24]. The DHS showed that lower education was associated with lower condom use rate among people having multiple partners. One possible explanation for this finding is that the result of the general population may not be applicable to the study population. This population differed by its religiosity level (though the religiosity level was not assessed in this survey). Meanwhile the high propensity to recommend condoms should be taken as a positive in increasing condom use among people with lower education. Interestingly, and encouragingly, for future perspectives, were the other results from the same analysis: 1) Information received at school was associated with a higher rate of condom recommendations; 2) Enhanced contact with PLHIV could lead to further commitment to condom use recommendations by religious leaders.

### Limitations of the Survey

This study had several limitations. The study examined the tendency of Christian religious leaders to supporting HIV prevention, based primarily on the sensitive topic of condom use. Raising issues related to STI and HIV among religious leaders is highly sensitive in traditionally religious countries. This was evidenced by 16 (6.9%) respondents failing to answer the question regarding having counseled condom use in the past. To limit response bias we used a self-administered questionnaire which gave the interviewee the opportunity to respond or not. A limitation of the self-administered questionnaire was that it was not possible to document some issues deeply. Another limitation was the fact that we did not document condom use by religious leaders. This survey shows a representation of Christian leaders in Antsirabe but we cannot assume that the results could be extrapolated to the rest of the country, or to the 92 other denominations present in Madagascar. We failed to investigate the training content that a few leaders received which might have helped in understanding how far behavioral change and HIV/AIDS education during sermons were the result of training, or related to the personality of leaders.

## Conclusion

The extent, and the ways in which, religious leaders engage in the fight against AIDS varies considerably among countries and denominations. Christian religious groups in Madagascar are being challenged to promote a clear and consistent response to the HIV/AIDS epidemic in agreement with international recommendations. They are willing to help but their messages remain mostly restricted to church doctrines with infrequent condom use counseling. Further enhancement of knowledge, involvement and interaction with people at risk, and the commitment of religious leaders to international HIV/AIDS recommendations on condom use are crucial if Christian religious leaders are to become key players in HIV/AIDS prevention within the Malagasy community.

## Supporting Information

Table S1 List of Christian denominations enrolled in the study and rate of condom recommendation.
(DOCX)

## References

[1] Kanda K, Jayasinghe A, Silva KT, Priyadarshani NG, Delpitiya NY, et al (2013) Religious leaders as potential advocates for HIV/AIDS prevention among the general population in Sri Lanka. Glob Public Health 8: 159–173 10.1080/17441692.2012.745892 23205515

[2] Trinitapoli J (2011) The AIDS-related activities of religious leaders in Malawi. Glob Public Health 6: 41–55. 923062350 [pii]; doi:10.1080/17441692.2010.486764. 10.1080/17441692.2010.486764 PMC2941704 20552476

[3] Green EC (2013) Rethinking AIDS prevention: Learning from successes in developing countries. Westport: Greenwood Publishing Group.

[4] Liebowitz J, Noll S (2006) The role of religion in educating Ugandan youth about HIV/AIDS. In: Morisky, DE., editor. Overcoming AIDS: Lessons learned from Uganda. In: Charlotte, NC: Information Age Publishing. 209–224.

[5] Manglos ND, Trinitapoli J (2011) The third therapeutic system: faith healing strategies in the context of a generalized AIDS epidemic. J Health Soc Behav 52: 107–122. 52/1/107 [pii]; doi:10.1177/0022146510395025. 10.1177/0022146510395025 PMC4015184 21362615

[6] Szaflarski M, Ritchey PN, Jacobson CJ, Williams RH, Baumann GA, et al.. (2013) Faith-Based HIV Prevention and Counseling Programs: Findings from the Cincinnati Census of Religious Congregations. AIDS Behav. doi:10.1007/s10461-013-0455-7. 10.1007/s10461-013-0455-7 PMC3682223 23568226

[7] Trinitapoli J (2009) Religious teachings and influences on the ABCs of HIV prevention in Malawi. Soc Sci Med 69: 199–209. S0277-9536(09)00244-5 [pii]; doi:10.1016/j.socscimed.2009.04.018. 10.1016/j.socscimed.2009.04.018 19447536

[8] SE CNLS (2007) Plan d’Action de Madagascar pour une lutte efficace contre le VIH/Sida 2007–2012. Available: http://www.aidstar-one.com/sites/default/files/prevention/resources/national_strategic_plans/Madagascar_2007-1012_French.pdf. Accessed 2009 July 02.

[9] Feldblum PJ, Nasution MD, Hoke TH, Van DK, Turner AN, et al.. (2007) Pregnancy among sex workers participating in a condom intervention trial highlights the need for dual protection. Contraception 76: 105–110. S0010-7824(07)00196-5 [pii]; doi:10.1016/j.contraception.2007.04.009. 10.1016/j.contraception.2007.04.009 17656179

[10] Randrianasolo B, Swezey T, Van DK, Khan MR, Ravelomanana N, et al.. (2008) Barriers to the use of modern contraceptives and implications for woman-controlled prevention of sexually transmitted infections in Madagascar. J Biosoc Sci 40: 879–893. S0021932007002672 [pii]; doi:10.1017/S0021932007002672. 10.1017/S0021932007002672 PMC3390975 18198005

[11] Khan MR, Rasolofomanana JR, McClamroch KJ, Ralisimalala A, Zafimanjaka MG, et al (2008) High-risk sexual behavior at social venues in Madagascar. Sex Transm Dis 35: 738–745 10.1097/OLQ.0b013e3181724383 18496471 PMC5824630

[12] Meekers D, Silva M, Klein M (2006) Determinants of condom use among youth in Madagascar. J Biosoc Sci 38: 365–380. S0021932005007200 [pii]; doi:10.1017/S0021932005007200. 10.1017/S0021932005007200 16613621

[13] Rahamefy OH, Rivard M, Ravaoarinoro M, Ranaivoharisoa L, Rasamindrakotroka AJ, et al (2008) Sexual behaviour and condom use among university students in Madagascar. SAHARA J 5: 28–35. 18496617 10.1080/17290376.2008.9724899

[14] WHO (2009) Position statement. Available http://www.who.int/hiv/pub/condoms/20090318_position_condoms.pdf?ua=1. Accessed 2014 April 18.

[15] Lanouette NM, Noelson R, Ramamonjisoa A, Jacobson S, Jacobson JM (2003) HIV- and AIDS-related knowledge, awareness, and practices in Madagascar. Am J Public Health 93: 917–919. 12773354 10.2105/ajph.93.6.917 PMC1447869

[16] Behets F, Andriamiadana J, Rasamilalao D, Ratsimbazafy N, Randrianasolo D, et al.. (2001) Sexually transmitted infections and associated socio-demographic and behavioural factors in women seeking primary care suggest Madagascar’s vulnerability to rapid HIV spread. Trop Med Int Health 6: 202–211. tmi690 [pii]. 10.1046/j.1365-3156.2001.00690.x 11299037

[17] Frickmann H, Schwarz NG, Girmann M, Hagen RM, Poppert S, et al (2013) Serological survey of HIV and syphilis in pregnant women in Madagascar. Trop Med Int Health 18: 35–39 10.1111/tmi.12007 23094758

[18] Kruse N, Behets FM, Vaovola G, Burkhardt G, Barivelo T, et al (2003) Participatory mapping of sex trade and enumeration of sex workers using capture-recapture methodology in Diego-Suarez, Madagascar. Sex Transm Dis 30: 664–670 10.1097/01.OLQ.0000079523.04451.8200007435-200308000-00014pii 12897692

[19] Leutscher P, Jensen JS, Hoffmann S, Berthelsen L, Ramarakoto CE, et al.. (2005) Sexually transmitted infections in rural Madagascar at an early stage of the HIV epidemic: a 6-month community-based follow-up study. Sex Transm Dis 32: 150–155. 00007435-200503000-00003 [pii]. 10.1097/01.olq.0000152820.17242.17 15729151

[20] Xueref S, Holianjavony J, Daniel R, Kerouedan D, Fabry J, et al.. (2003) The absence of HIV seropositivity contrasts with a high prevalence of markers of sexually transmitted infections among registered female sex workers in Toliary, Madagascar. Trop Med Int Health 8: 60–66. 986 [pii]. 10.1046/j.1365-3156.2003.00986.x 12535252

[21] Stoebenau K, Hindin MJ, Nathanson CA, Rakotoarison PG, Razafintsalama V (2009) “… But then he became my sipa”: the implications of relationship fluidity for condom use among women sex workers in Antananarivo, Madagascar. Am J Public Health 99: 811–819. 19299685 10.2105/AJPH.2007.118422 PMC2667855

[22] Harijaona V, Ramambason JD, Morisset R, Rasamindrakotroka A, Ravaoarinoro M (2009) Prevalence of and risk factors for sexually-transmitted infections in hidden female sex workers. Med Mal Infect 39: 909–913. S0399-077X(09)00032-8 [pii]. 10.1016/j.medmal.2009.01.007 19269760

[23] Leutscher PD, Behets F, Rousset D, Ramarokoto CE, Siddiqi O, et al.. (2003) Sexual behavior and sexually transmitted infections in men living in rural Madagascar: implications for HIV transmission. Sex Transm Dis 30: 262–265. 00007435-200303000-00017 [pii]. 10.1097/00007435-200303000-00017 12616148

[24] DHS Madagascar (2008–2009), Demographic and Health Survey. Available http://dhsprogram.com/what-we-do/survey/survey-display-296.cfm.

[25] Bangré H (2004) La France a transfusé l’Afrique avec du sang contaminé. Accessed 10 april 2014; Available: http://www.afrik.com/article7614.html Accessed 2014 April 10.

[26] Benton KW (2008) Saints and sinners: training Papua New Guinean (PNG) Christian Clergy to respond to HIV and AIDS using a model of care. J Relig Health 47: 314–325 10.1007/s10943-007-9158-6 19105022

[27] Kagimu M, Kaye S, Ainomugisha D, Lutalo I, Walakira Y, et al (2012) Evidence-based monitoring and evaluation of the faith-based approach to HIV prevention among Christian and Muslim youth in Wakiso district in Uganda. Afr Health Sci 12: 119–128 10.4314/ahs.v12i2.7jAFHS.v12.i2.pg119pii 23056016 PMC3462538

[28] Watt MH, Maman S, Jacobson M, Laiser J, John M (2009) Missed opportunities for religious organizations to support people living with HIV/AIDS: findings from Tanzania. AIDS Patient Care STDS 23: 389–394 10.1089/apc.2008.0195 19335171 PMC3521158

[29] Zou J, Yamanaka Y, John M, Watt M, Ostermann J, et al.. (2009) Religion and HIV in Tanzania: influence of religious beliefs on HIV stigma, disclosure, and treatment attitudes. BMC Public Health 9: 75. 1471-2458-9-75 [pii]; doi:10.1186/1471-2458-9-75. 10.1186/1471-2458-9-75 PMC2656538 19261186

