# Supplementary material for: Can Churches Play a Role in Combating the HIV/AIDS Epidemic? A Study of the Attitudes of Christian Religious Leaders in Madagascar
Source: PLoS One. 2014 May 13;9(5):e97131. doi: 10.1371/journal.pone.0097131 (PMC4019665; doi:10.1371/journal.pone.0097131)
Supplement: Table S1 — List of Christian denominations enrolled in the study and rate of condom recommendation. (DOCX) [file pone.0097131.s001.docx]

Table 1: List of Malagasy Christian denominations and of religious leaders enrolled in the study

| Malagasy Church* | English name | Leaders included | Will  Recommend Condom use.** | |
| --- | --- | --- | --- | --- |
|  |  | N=231 | N=91 | (%) |
| Fiagonana Loterana malagasy | Malagasy Lutherian Church | 21 | 8 | (38.1) |
| Eglizy katolika romana | Roman Catholic Church | 21 | 11 | (52.4) |
| Fiangonan’Andranovisy | Rhema | 15 | 3 | (20.0) |
| Jesosy Mamonjy | Savior Jesus | 15 | 3 | (20.0) |
| Fiangonana advantista mitandrina ny andro fahafito | Seventh-Day Adventist Church | 12 | 4 | (33.3) |
| Fiangonana fifohazana Fanahy masina eto Madagasikara | Holy Spirit Revival Church of Madagascar | 12 | 12 | (100.0) |
| Fiangonan’I Jehova Tompon’ny Sabata | Church of Jehovah, the Setter of Sabbath | 10 | 3 | (30.0) |
| Fiangonana Protestanta Vaovao eto Madagasikara | New Protestant Church of Madagascar | 10 | 3 | (33.3) |
| Fiangonan’I Jesosy Kristy eto Madagasikara | Jesus Christ Church of Madagascar | 9 | 4 | (44.4) |
| Zanaka Mpampiavana | Sons of Reconciliation | 9 | 1 | (11.1) |
| Fiangonana anglikana | Anglican Church | 8 | 5 | (62.5) |
| Fiangonana Apokalipsy | Church of The Revelations | 8 | 3 | (37.5) |
| Assemblée de Dieu | God’s Assembly | 7 | 5 | (71.4) |
| Fiangonan’ny fahasoavana | Grace Church | 7 | 1 | (14.3) |
| Fiangonana malagasy tranozozoro antranobiriky | Malagasy Church of Reed and Bricks | 6 | 1 | (16.7) |
| Misiona Evanjelika Teratany eto Madagasikara | Native Evangelic Mission of Madagascar | 6 | 5 | (83.3) |
| Fiangonana batista biblika | Biblical Baptist Church | 6 | 1 | (16.7) |
| Vavolombelon’i Jehova | Jehovah’s Witnesses | 6 | 2 | (33.3) |
| Jesosy fanekena vaovao | Yawoshua New Convenant | 6 | 1 | (20.0) |
| Fiangonan’ny olomasin’ny andro farany | [The Church of Jesus Christ of Latter-day Saints](http://ccs.infospace.com/ClickHandler.ashx?du=www.lds.org&ru=http%3a%2f%2fwww.lds.org%2f%3flang%3deng&ld=20140124&ap=2&app=1&c=bechiro.row&s=bechiro&coi=771&cop=main-title&euip=41.204.106.84&npp=2&p=0&pp=0&pvaid=6c206442d61340a1af129053db10ed6a&ep=2&mid=9&en=2zY3AxucHTWIlwtCfSS5byX4irrsuae5QScQEViHEF8%3d&hash=0C286E31B22C345581686193A78B39E7) | 6 | 1 | (16.7) |
| Fiangonana batista fiainam-baovao | New Life Baptist Church | 5 | 4 | (80.0) |
| Fiangonana aram-pilazantsara | Evangelical Church | 5 | 2 | (40.0) |
| Fiangonan’ny fifohazan’ny mpianatry ny Tompo | Revival Church of Jesus’ Disciples | 5 | 1 | (20.0) |
| Denomination unkown | Denomination unkown | 4 | 3 | (75.0) |
| Jesosy fandresena | Jesus Winner | 3 | 1 | (33.3) |
| Pantekotista mitambatra | Union of the Pentecostals | 3 | 1 | (33.3) |
| Communauté de l’épouse du Christ | Jesus’ Wife Community | 3 | 1 | (33.3) |
| Vahao ny oloko | Free my People | 2 | 1 | (50.0) |
| Fiangonana batista Mamorivokatra | Baptist Church of Mamorivokatra | 1 | 0 | (0.0) |

*4 did not answer the question about denomination, **16 did not answer this question
